# Progress in research on cognitive frailty in the older adults: a narrative review

**DOI:** 10.3389/fmed.2026.1791816

**Published:** 2026-06-03

**Authors:** Shanshan Shao, Binyan Zhao, Shixiao Jin, Liyan Sha

**Affiliations:** The Second Affiliated Hospital of Dalian Medical University, Dalian, China

**Keywords:** assessment tools, cognitive frailty, influencing factors, older adults, review

## Abstract

With the extension of average life expectancy and the intensification of the global aging trend, cognitive frailty in the older adults has become a new concept and a research hotspot in the field of geriatrics. Cognitive frailty is somewhat reversible in the older adults, and through early screening and intervention, it is possible to effectively restore or delay the decline of cognitive function, thereby preventing progression to dementia, which has become an important strategy for achieving healthy aging. This article will provide a comprehensive review of the concept of cognitive frailty, its epidemiological status, assessment tools, influencing factors, and intervention strategies, aiming to deepen medical workers' understanding of cognitive frailty in the older adults and promote the diagnosis, treatment, and prevention of cognitive frailty.

## 1 Introduction

**Figure 1:**
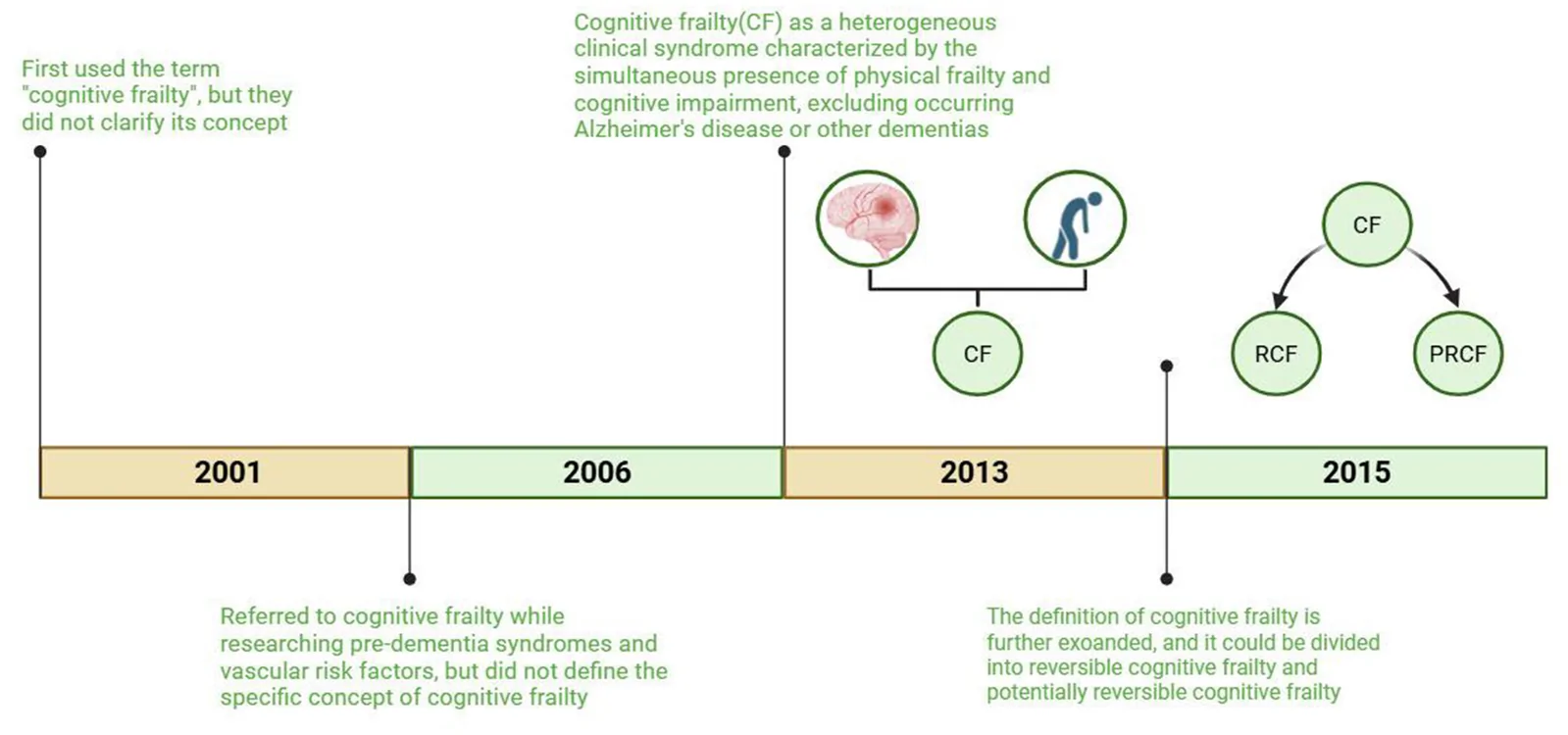
The development process and definition of cognitive frailty.

In 2001, American scholar Paganini-Hill et al. (1) first used the term “cognitive frailty” (Cognitive frailty, CF) when studying the risk factors for Alzheimer's disease using the “drawing test,” although they did not clarify its concept; in 2006, Panza F et al. (2) also referred to cognitive frailty while researching pre-dementia syndromes and vascular risk factors, but similarly did not define the specific concept of cognitive frailty. It was not until 2013 that an international consensus group formed by the International Society for Nutrition and Aging and the International Association of Gerontology and Geriatrics (3) defined cognitive frailty as a heterogeneous clinical syndrome characterized by the simultaneous presence of physical frailty and cognitive impairment, excluding occurring Alzheimer's disease or other dementias. In 2015, Ruan et al. (4) proposed that cognitive frailty could be divided into reversible cognitive frailty and potentially reversible cognitive frailty. The diagnostic criteria for reversible cognitive frailty (Reversible Cognitive Frailty, RCF) include the presence of subjective cognitive decline (Subjective Cognitive Decline, SCD) and/or positive biological markers of amyloid beta accumulation and neurodegenerative changes at the same time as physical frailty; the diagnostic criteria for potentially reversible cognitive frailty (Potential Reversible Cognitive Frailty, PRCF) require the presence of mild cognitive impairment while excluding Alzheimer's disease or other types of dementia. This classification has important guiding significance for the judgment of disease prognosis and the selection of intervention strategies. The development process and definition of cognitive frailty are shown in Figure 1.

The prevalence of cognitive frailty in the older adults exhibits significant differences across different populations. According to existing studies, the overall prevalence of cognitive frailty ranges widely, with a prevalence amongst the older adults abroad being 1%−39.9%, while in China it is observed to be 2.4%−50.1%(5, 6), indicating that the situation in our country is relatively complex. Research by Xie et al. (7) found that in China, the prevalence of cognitive frailty among community-dwelling older adults was 14.3%; in nursing homes, the prevalence was 22.7%; and among hospitalized patients, this proportion reached 25.2%. Relevant consensus suggests that the overall prevalence of cognitive frailty in our country is between 5.0 and 18.0% (8). Although the range of prevalence proposed in the consensus is lower, actual research results show that the prevalence of cognitive frailty among the older adults in our country is generally high, especially among hospitalized and nursing home populations. The incidence of cognitive impairment shows significant differences across different studies, which may stem from variations in diagnostic criteria (such as different scale selections), geographical settings (regional differences), and research environments (such as clinical *vs*. community settings).

## 2 Pathophysiological mechanisms of cognitive frailty

**Figure 2:**
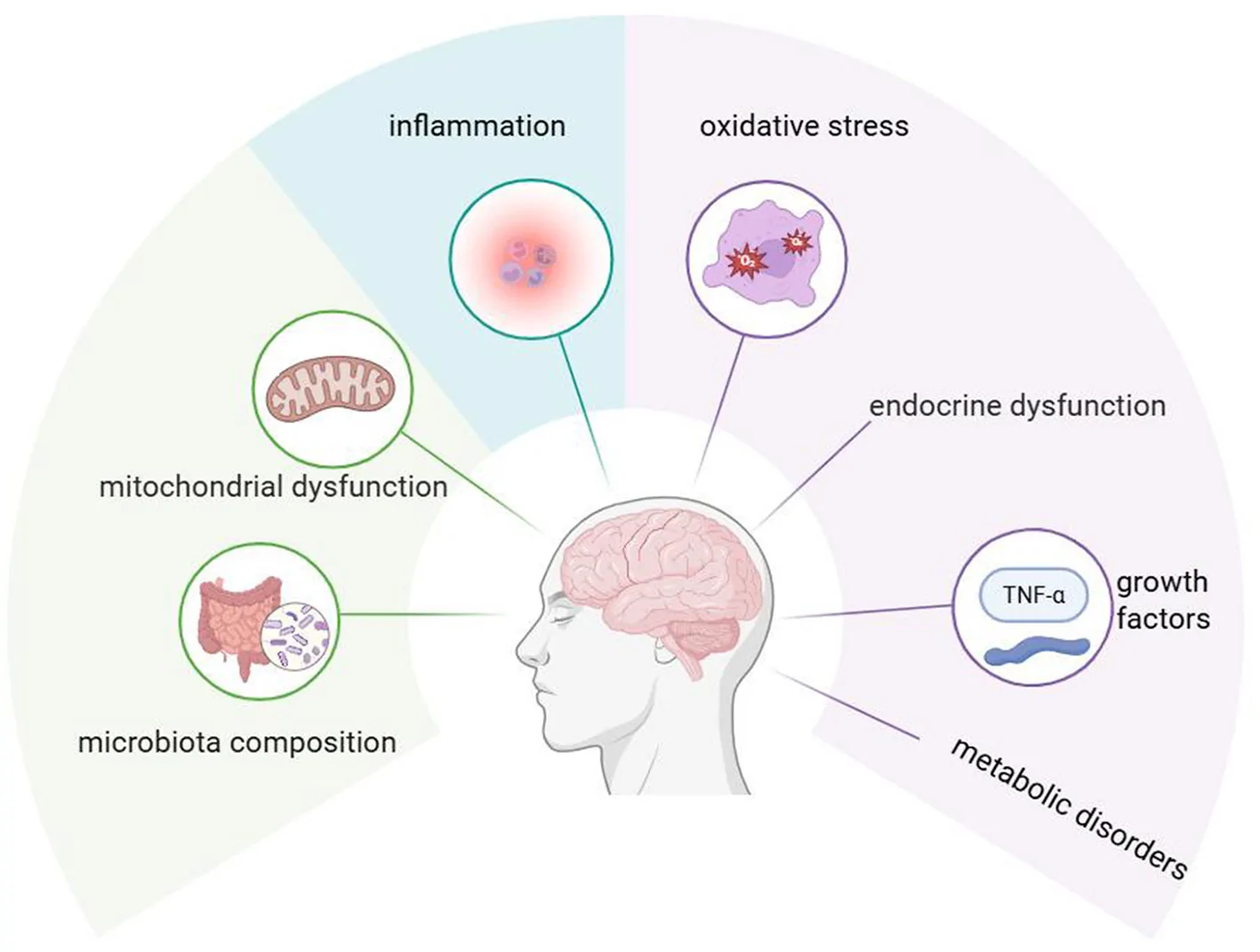
The specific mechanisms are detailed as follows, with the mechanism diagram.

**Figure 3:**
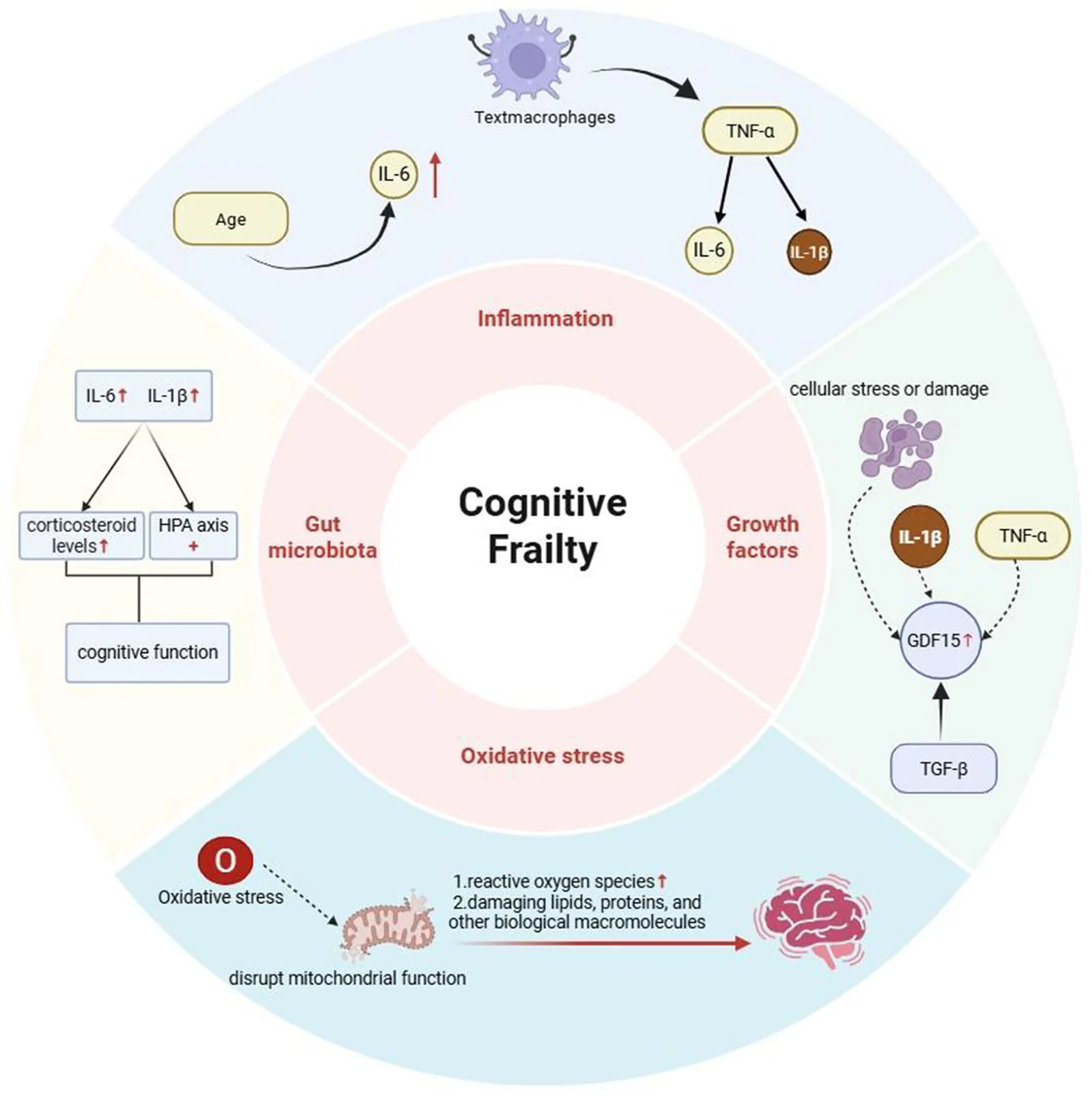
The specific mechanisms are detailed as follows, with the mechanism diagram.

Cognitive frailty results from the joint action of genes and environmental factors (9, 10). The potential mechanisms mainly include inflammation, oxidative stress, metabolic disorders, endocrine dysfunction, mitochondrial dysfunction, stem cell/growth factor, and changes in microbiota composition (11, 12). The specific mechanisms are detailed as follows, with the mechanism diagram shown in Figures 2, 3.

### 2.1 Inflammation and growth factors

Chronic inflammation is characterized by persistently elevated inflammatory cytokines and is a potential biological mechanism related to cognitive decline and physical frailty, thus considered a key driving factor for the development of cognitive frailty (13–15). Interleukin-6 (IL-6) is the most important cytokine in inflammation; serum IL-6 levels increase with age and are associated with declines in physical function (slow walking speed and reduced muscle strength) and cognitive function (12). Tumor necrosis factor-alpha (TNF-α), primarily secreted by macrophages, acts as an early pro-inflammatory cytokine that stimulates the generation of other cytokines, including interleukin-1 beta (IL-1β) and IL-6 (16), the latter produced by various cells and playing a critical role in the transition from acute to chronic inflammation (17), inducing the liver to produce acute phase proteins such as C-reactive protein (CRP). Growth differentiation factor 15 (GDF15), belonging to the transforming growth factor-beta (TGF-β) family, is induced by cellular damage and metabolic stress and is considered a sensitive marker of mitochondrial dysfunction and biological aging (18–20); it is upregulated due to cellular stress or damage and can be induced by various growth factors and cytokines, including TGF-β, TNF-α, and IL-1β. Research in the field of cognitive frailty indicates significant correlations between CRP and TNF-α with cognitive frailty, further supporting their potential as biomarkers for clinical application; GDF15 also shows an association with cognitive frailty. Moreover, although studies on soluble TNF-alpha receptor type II (sTNF-RII) and human high-temperature requirement serine protease A1 (HTRA1) are relatively few, they have demonstrated significant and potential associations (21). These findings provide a basis for incorporating sTNF-RII and HTRA1 into subsequent research, helping to reveal the inflammatory mechanisms related to cognitive frailty further. It can be seen that by early identification of individuals with elevated IL-6 levels and timely intervention (such as anti-inflammatory treatment), it may be possible to reduce the risk of cognitive frailty.

### 2.2 Oxidative stress

With advancing age, the balance between oxidation and antioxidation is disrupted, leading to decreased antioxidant capacity and increased oxidative stress damage, resulting in elevated levels of lipid peroxidation products that promote cognitive impairment (22). Oxidative stress damage can disrupt mitochondrial function, increasing levels of reactive oxygen species (ROS), damaging lipids, proteins, and other biological macromolecules, and compromising brain tissue structure, ultimately affecting cognitive function (23). Specific exercises, such as Ba Duan Jin, have been shown to have both antioxidant and anti-inflammatory effects, which can improve cognitive frailty (24).

### 2.3 Gut microbiota

The primary factors influencing cognitive frailty are largely associated with alterations in gut microbiota composition (25). Gut microbiota modulate brain and intestinal functions through the hypothalamic-pituitary-adrenal (HPA) axis. The HPA axis, a crucial component of the neuroendocrine system, participates in stress responses. Chronic stress may compromise the intestinal barrier, leading to dysbiosis. This dysbiosis induces bacterial translocation and immune system activation, increasing levels of Clostridium difficile while elevating inflammatory cytokines IL-6 and IL-1β. This sequence results in HPA axis hyperactivation and elevated corticosteroid levels. Corticosteroids can cross the blood-brain barrier, affecting hippocampal and amygdala structures, thereby further impacting cognitive function (26, 27). Gut microbiota can also influence brain behavior, memory, and cognitive function through pathways involving neurotransmitters, the vagus nerve, and immune regulation (28). For dysbiosis, cognitive function can be improved mainly by regulating the composition of gut microbiota, such as through probiotics supplementation.

## 3 Cognitive frailty assessment tools

Currently, the mainstream approach to measuring cognitive frailty involves using combined physical frailty and cognitive function assessments to screen older adults individuals for cognitive frailty. Among physical frailty assessments, the Fried Frailty Phenotype (FFP) is most commonly employed. The FFP was proposed by Fried et al. (29) in 2001 based on the US Cardiovascular Study. It comprises five assessment indicators: involuntary weight loss, self-reported fatigue, physical activity level, walking speed, and grip strength. Meeting any 1–2 criteria indicates pre-frailty, while meeting three or more defines frailty. With its limited number of objective, quantitative indicators, the FFP serves as a broad-spectrum tool for evaluating physical frailty (30). The Clinical Practice Guidelines for Frailty Management in the Asia-Pacific Region (31) indicate that this scale effectively predicts mortality, disability, falls, hospitalization, and surgical risks in older adults. It is widely applied in assessing physical frailty among the older adults population in China.

Common tools for assessing cognitive function in older adults include the Mini-Mental State Examination (MMSE) and the Montreal Cognitive Assessment (MoCA). Nasreddine (32) compared the applicability of MMSE and MoCA for screening mild cognitive impairment (MCI) in older adults, finding that MoCA demonstrated higher MCI detection rates than MMSE. Chinese researchers Chen Xi et al. (33) concluded that combining MoCA with the Fried Scale offers ideal sensitivity and specificity for cognitive frailty screening. This approach is operationally simple, time-efficient, and suitable for large-scale screening. Therefore, the Fried Frailty Phenotype and MoCA are recommended for assessing cognitive frailty.

## 4 Influencing factors of cognitive frailty

In the older adults population, the development of cognitive frailty is a complex process influenced by multiple factors, including demographic characteristics, lifestyle habits, nutritional status, and psychological and social conditions. Currently, the etiology and pathological mechanisms of cognitive frailty in the older adults remain unclear. The cognitive frailty model proposes that factors affecting individual health encompass not only individual and disease-related factors but also psychological and social factors.

### 4.1 Demographic characteristics

Research indicates that sex, age, educational attainment, marital status, living arrangements, and coexisting chronic conditions are associated with cognitive frailty. Studies suggest a higher prevalence of cognitive frailty among older adults women compared to men. South Korean researchers Kim M et al. (34) found cognitive impairment rates of 2.8 and 3.8% among older adults men and women, respectively. This indicates a higher prevalence among older adults women, consistent with findings by Solfrizzi V, Sharma M, and Chinese scholars Liu J and Zhai Yujia (35–38). Compared to men, older women face a higher risk of cognitive frailty. This may be associated with reduced estrogen levels after menopause, leading to vitamin D deficiency and diminished neuroprotective effects, thereby impacting neuromuscular balance and brain neural activity. Age has long been recognized as the most significant independent risk factor for cognitive impairment and dementia. Solfrizzi V et al. (35, 39) utilized data from the Italian Longitudinal Study on Aging to demonstrate that the occurrence of cognitive frailty correlates with age. The prevalence of cognitive frailty increases with advancing age. For instance, Xie et al. (40) studied 1,585 older adults individuals and found that the prevalence of cognitive frailty was 3.48% in the 75–80 age group, 7.22% in the 81–85 age group, and 15.98% in those aged ≥86 years. However, the age effect may be due to the combined influence of factors such as the accumulation of complications, polypharmacy, and functional decline, rather than age itself. A study of Malaysian older adults individuals demonstrated a significant association between educational attainment and cognitive frailty (41). Ren Ying et al. (42) surveyed 438 older adults individuals from five medical-care-integrated nursing homes in Anhui Province, finding that those with higher educational attainment exhibited lower rates of cognitive frailty. Older adults individuals with higher education can effectively delay cognitive and physiological frailty by enhancing cognitive reserves, improving health literacy, and promoting healthy behaviors, thereby reducing the risk of cognitive frailty. In the context of comorbid chronic diseases, particularly those affecting the cardiovascular and cerebrovascular systems (such as hypertension, diabetes, heart failure, and stroke), these conditions represent major risk factors for cognitive frailty. These diseases may accelerate brain aging and dysfunction through mechanisms including impaired blood supply and inflammation. Additionally, marital status and living arrangements significantly influence cognitive frailty occurrence. A cross-sectional survey of 5,708 community-dwelling older adults in China revealed that being unmarried/widowed and residing in rural areas constitute risk factors for cognitive frailty among the older adults (43). Within the chronic disease domain, Hou Wenwen et al. (44) found a higher prevalence of cognitive frailty among patients with chronic kidney disease (CKD). A survey by Zhang Yajuan et al. (45) among older adults patients with type 2 diabetes and hypertension revealed a cognitive frailty incidence rate of 30.70%, significantly higher than in patients with diabetes alone. Yang Zhen and Zhang Huijun's (46) study similarly indicated higher prevalence among community-dwelling older adults with chronic diseases. Most studies have focused solely on the number and types of chronic diseases, without taking specific medications into detailed account; the independent effects of these medications on cognitive and physical function have been well established (47).

### 4.2 Lifestyle and behavioral habits

Physical exercise, smoking, alcohol consumption, and sleep duration are all associated factors influencing cognitive frailty in older adults. Some international studies have identified low physical activity as a risk factor for cognitive frailty (48). Esteban-Cornejo et al. (49) recruited 3,677 community-dwelling older adults in Spain, collecting self-reported physical activity and cognitive frailty status data. Results indicated that engaging in physical activities enhances the functional capacity of cognitively declining older adults. Murukesu R et al. (50) demonstrated through a Malaysian intervention study that cognitive frailty correlates with physical activity levels, recommending that cognitively impaired seniors maintain physical engagement. Data from the Chinese Comprehensive Geriatric Assessment Survey (CCGAS) indicates that physical activity volume is an independent risk factor for cognitive frailty—lower activity levels correlate with higher cognitive impairment risk (25). Lu et al. (51) demonstrated that increased physical exercise can effectively delay the progression of cognitive frailty. Zhao X. R. et al. (52) found that individuals with histories of smoking and alcohol consumption face higher risks of cognitive frailty. The aforementioned studies examined only past smoking and alcohol consumption; they did not assess current smoking or alcohol consumption, nor did they quantify cumulative exposure (pack-years or drink units). The toxic chemicals in cigarettes induce chronic inflammation, oxidative stress, and DNA methylation, thereby impairing physical function (53). Alcohol consumption interferes with DNA repair, while its metabolites directly damage cells, increasing the risk of aging and disease (54). Zhao Y et al. (55), utilizing data from the Western China Health and Aging Trend Survey, found that prolonged sleep duration is associated with cognitive frailty in older adults. Adequate and high-quality sleep is equally crucial for preventing cognitive frailty. Sleep deprivation increases the risk of cardiovascular and cerebrovascular diseases, thereby indirectly impacting cognitive health.

### 4.3 Nutritional status

Malnutrition may accelerate cognitive decline by impairing neuronal function, brain metabolism, and immune function (56). Zhu et al. (57) demonstrated a significant association between cognitive impairment and malnutrition, with higher prevalence rates observed in community settings compared to hospitals. Jia et al. studied 1,086 individuals aged 65 and older in Shandong and Chongqing, finding that unsaturated fatty acids in fish and shellfish, along with anti-inflammatory and antioxidant compounds in fruits, offer protective effects against cognitive frailty. Thus, increasing intake of fish, shellfish, and fruits while incorporating muscle-strengthening exercises may reduce the incidence of cognitive frailty (58). Cross-sectional studies cannot establish a causal relationship between malnutrition and cognitive decline.

### 4.4 Mental health factors

Mental health conditions such as depression and anxiety are closely associated with cognitive frailty. Depression may not only directly impact brain function but also indirectly affect cognitive health by reducing social interaction and physical activity. While international research on the influence of mental health on cognitive frailty is limited, existing studies suggest that mental health impacts both the level of cognitive frailty and quality of life in older adults. Navarro-Pardo E et al. (48) found in a cross-sectional study that older adults with mental health issues were more prone to cognitive frailty. An Italian cross-sectional survey of individuals aged 65 and older revealed a higher prevalence of depressive tendencies among those experiencing cognitive frailty, suggesting a link between cognitive frailty and mental health in older adults (35). Similarly, Rivan N et al. (41) in Malaysia identified depression as an independent risk factor for cognitive frailty. Ma L et al. (47) assessed depression in older adults using the Geriatric Depression Scale (GDS), confirming depression as an independent risk factor for cognitive frailty with a positive correlation between the two. Findings from Kwan R, Wang C, Wang J et al. (59–61) aligned with MaL's results. However, some studies indicate that the association between psychological factors and cognitive frailty depends on specific types. De Roeck EE et al. (62) found that cognitive frailty was associated with symptoms of mood disorders but not with loneliness.

### 4.5 Social environmental factors

Lack of social support increases psychological stress and reduces cognitive stimulation, constituting another significant risk factor (63). A Malaysian study identified insufficient social support as a risk factor for cognitive frailty (41). De Roeck EE et al. (62), based on a Belgian cross-sectional study, found a negative correlation between social support and cognitive frailty. Wang C et al. (60) investigated older adults aged 60–89 in Shanghai's Pudong New Area. After controlling for sex, age, marital status, chronic diseases, BMI, medication use, smoking, alcohol consumption, physical exercise, and mental health, they found social support to be an independent protective factor against cognitive frailty. Zhao D et al. (64), using the Shandong Older Adults Health Cohort, found a negative correlation between cognitive frailty and participation in social activities, suggesting that older adults without cognitive frailty are more likely to engage in social activities. In summary, in clinical practice, physical exercise, nutritional status, and mental health are considered operational intervention factors.

## 5 Interventions for cognitive frailty

Interventions for cognitive frailty worldwide include dietary interventions, exercise interventions, cognitive training interventions, dual-task training interventions, neuromodulation interventions, and hyperbaric oxygen therapy (65). Cognitive frailty exhibits reversible or dynamic characteristics, providing a time window for early intervention. Existing intervention studies, particularly domestic ones, pre=dominantly focus on exercise interventions (66–70), lacking multidimensional, personalized, and long-term follow-up evidence. However, clinical settings urgently require healthcare institutions to adopt targeted intervention strategies tailored to the domestic medical environment and patient conditions to improve cognitive frailty and enhance cognitive function in the older adults.

### 5.1 Dietary intervention

Regarding dietary interventions, Devranis et al. (71) compared the effects of the Mediterranean diet, ketogenic diet, and MIND diet on preventing cognitive decline in older adults. They found adherence to the Mediterranean diet yielded the greatest cognitive benefits. Its abundance of vegetables, fruits, fish, whole grains, legumes, and olive oil—rich in healthy monounsaturated fatty acids, dietary fiber, and antioxidant nutrients-can enhance cognitive and physical functioning by increasing anti-inflammatory omega-3 polyunsaturated fatty acids. Research findings by Ye Ming and Li Shuguo (72) indicate that supplementation with enteral nutritional suspensions can improve nutritional status and cognitive impairment in hospitalized patients with cognitive frailty. However, the study had a small sample size and examined only a limited number of indicators, so further research is needed to validate these findings. Nutritional intervention alone has limited effects on improving overall cognitive function in individuals with cognitive frailty; it should be combined with other intervention methods.

### 5.2 Dual-task training intervention

Kwan et al. (73) conducted dual-task training on 293 cognitively frail older adults individuals. The findings demonstrated that exercise-cognitive training effectively enhances cognitive function and reduces physical frailty in this population. In China, Han Jun et al. (74) divided 66 cognitively frail older adults participants into two groups, yielding results consistent with Kwan's study. However, this study did not assess the impact of adherence to the intervention on its effectiveness. Furthermore, all of the aforementioned studies suffer from the limitation of an insufficient follow-up period (≤ 6 months); follow-up should be extended to ≥12 months to assess rates of cognitive maintenance and reversal of cognitive decline. Therefore, dual-task training alone may not be sufficient to fully reverse cognitive decline.

### 5.3 Hyperbaric oxygen therapy

Hong Shanshan et al. (75) divided 56 older adults individuals with cognitive frailty into a hyperbaric oxygen group (26 cases) and a control group (30 cases) based on treatment modality. The hyperbaric oxygen group received 8 weeks of hyperbaric oxygen therapy (five sessions per week, 110 min per session), while the control group received routine health education. Following the intervention, the MoCA scores in the hyperbaric oxygen group improved compared to the control group, suggesting that hyperbaric oxygen therapy can reduce the degree of cognitive frailty in older adults patients. Currently, research on hyperbaric oxygen intervention for individuals with cognitive frailty remains limited, and the duration, frequency, and protocol of hyperbaric oxygen therapy still require extensive clinical trials to determine.

Building upon these interventions, we propose a multimodal integrated approach: the “PCNS” model combining physical exercise (P), cognitive training (C), nutritional support (N), and psychosocial intervention (S). Single-modality interventions yield limited effects in cognitive frailty, whereas multimodal integration may achieve superior outcomes. However, there are currently no direct, high-quality randomized controlled trials to validate the efficacy of the “PCNS” model in individuals with cognitive decline; therefore, the “PCNS” model remains in the hypothesis-generation phase and urgently requires validation through clinical trials. Building upon multimodal intervention, tailored intervention plans can be developed for older adults patients based on different subtypes (e.g., reversible and potentially reversible) and specific risk factors (e.g., inflammation levels, nutritional status). Future efforts should establish a comprehensive management model encompassing community screening, hospital diagnosis, home-based intervention, and long-term follow-up, with cost-effectiveness evaluation to form a full-cycle management pathway.

## 6 Challenges and future perspectives of the cognitive frailty

While existing assessment tools (e.g., FFP and MoCA scales) are widely used, they have limitations such as insufficient sensitivity, cultural dependency, and high subjectivity. Beyond questionnaire-based assessments, three blood biomarkers—GDF15, cinnamic acid, and nicotinamide—can detect cognitive frailty. Future research should integrate these biomarkers into evaluation tools for precise cognitive frailty detection. In neuroimaging, white matter hyperintensities correlate with cognitive decline, balance impairment, and gait disturbances; Volumetric reduction in hippocampal subregions shows significant correlation with cognitive frailty, making imaging data a viable assessment metric. Given the absence of localized cognitive frailty assessment tools in China, future research should focus on enriching evaluation indicators and developing a comprehensive assessment tool that integrates clinical scales, blood biomarkers, and neuroimaging features, tailored for primary healthcare settings in China. In routine geriatric medical practice, the three factors of physical exercise, nutritional status, and mental health are operationally relevant, based on three main reasons. First, these indicators can be quickly obtained through validated assessment tools. Second, related interventions are widely available and low-cost, such as home exercise programs, dietary consultations, and behavioral activation interventions targeting depressive and anxiety symptoms. Third, interventions addressing these factors have been shown to improve or prevent cognitive frailty in community and primary care settings. Therefore, we recommend prioritizing these three areas in clinical screening and intervention programs. With the deep integration of new-generation information technologies like the internet and artificial intelligence, digital technologies are increasingly applied in smart health and older adults care. Future intervention systems can incorporate digital health technologies—such as wearable devices (monitoring gait, activity levels, and sleep) and smartphone applications (delivering digital cognitive games)—to enable high-frequency, objective, dynamic monitoring and early warning of cognitive frailty. To address China's aging challenges, future research should focus on developing a comprehensive AI-integrated intervention system for cognitive frailty. Specifically, leveraging China's large older adults population and unique sociocultural context, large-scale prospective cohort studies empowered by AI should be conducted. This would establish a national-level cohort dedicated to older adults cognitive frailty, revealing the natural disease course, progression trajectories, and specific risk factors among Chinese older adults patients. Research dimensions should transcend the current limitations of hospital-centered cross-sectional studies. Leveraging AI-assisted tools such as mobile health and remote monitoring, we should delve into the cognitive frailty characteristics and personalized intervention strategies for high-risk groups, including rural left-behind seniors, those living alone, and older adults individuals with comorbidities. Given the scarcity of longitudinal studies, future research should prioritize longitudinal approaches. Regarding intervention pathways, actively explore the potential of traditional Chinese medicine therapies—such as acupuncture and herbal formulas—in improving cognitive frailty. Integrate traditional medical wisdom to develop personalized, scalable TCM constitution-based care protocols. Ultimately, by deeply integrating multi-source data through AI technology and spanning the entire “assessment-prediction-intervention” chain, we aim to develop comprehensive, efficient, and precise cognitive frailty intervention protocols tailored to the Chinese population. This will provide scientific support for slowing disease progression and enhancing the health status of the older adults.

## 7 Conclusion

Cognitive frailty is an emerging concept, and older adults patients are prone to physical frailty and cognitive impairments. This article summarizes the unmodifiable and modifiable factors associated with cognitive frailty in older adults patients, and based on this, it organizes the intervention measures related to cognitive frailty, proposing a comprehensive intervention model called “PCNS.” Finally, it offers actionable suggestions regarding the development direction of cognitive frailty. This review emphasizes the importance of the influencing factors and intervention measures of cognitive frailty, which is significant for early identification and intervention of cognitive frailty to reduce adverse outcomes. Inclusion and exclusion of the literature(see Supplementary material-Figure 4). Retrieval plan(see Supplementary material-Table 1). Summary of intervention measures(see Supplementary material-Table 2).
