# Correction: Progress in research on cognitive frailty in the older adults: a narrative review

**DOI:** 10.3389/fmed.2026.2015103

**Published:** 2026-10-13

**Authors:** Shanshan Shao, Binyan Zhao, Shixiao Jin, Liyan Sha

**Affiliations:** The Second Affiliated Hospital of Dalian Medical University, Dalian, China

**Keywords:** assessment tools, cognitive frailty, influencing factors, older adults, review

Authors Shanshan Shao and Binyan Zhao were erroneously omitted as equal contributing (co-first) authors. Shanshan Shao and Binyan Zhao contributed equally to this work.

The original version of this article has been updated.

