# Supplementary material for: Progress in research on cognitive frailty in the older adults: a narrative review
Source: Front Med (Lausanne). 2026 Jun 3;13:1791816. doi: 10.3389/fmed.2026.1791816 (PMC13271909; doi:10.3389/fmed.2026.1791816)
Supplement: Supplementary file 1 [file Data_Sheet_1.pdf]

Supplementary Material

1 Supplementary Data

2 Supplementary Figures and Tables

2.1 Supplementary Figures

Supplementary Figure 1.

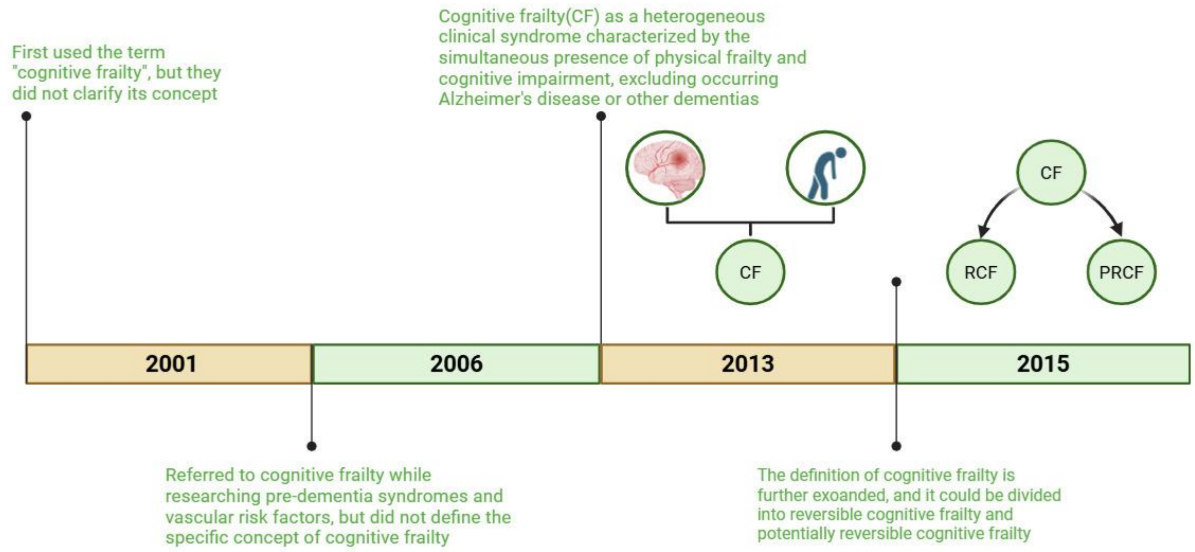

Supplementary Figure 2.

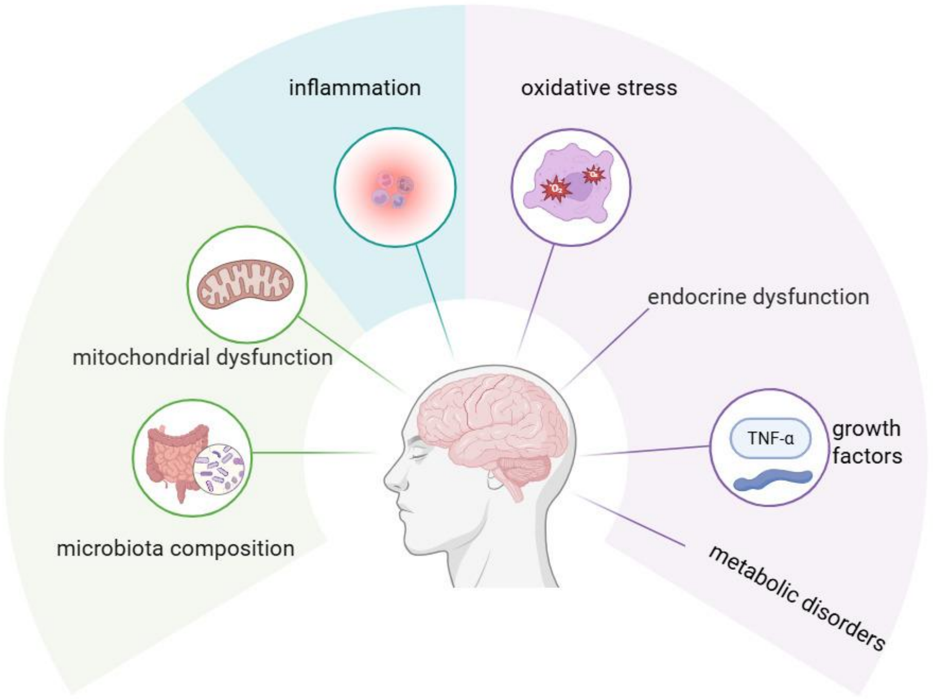

Supplementary Figure 3.

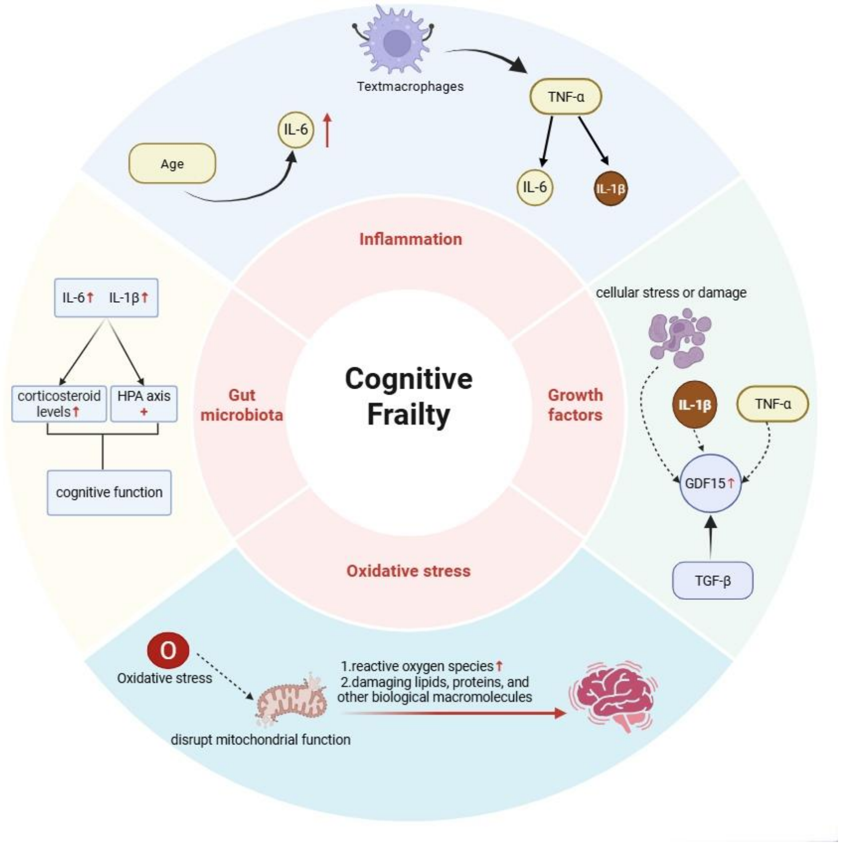

Supplementary Figure 4.

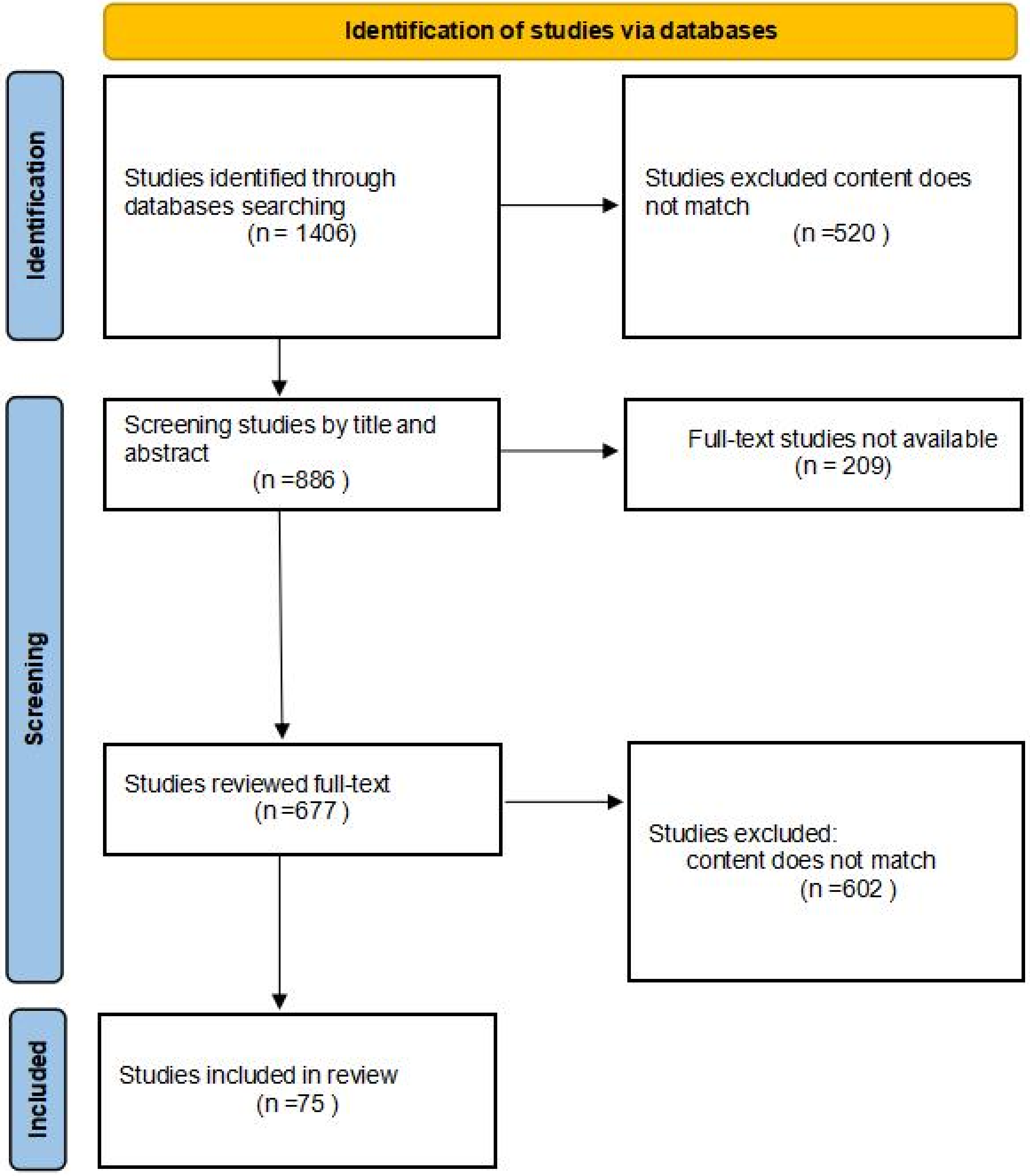

2.2 Supplementary Table 1.

This manuscript presents a narrative review on elderly cognitive frailty patients. The two authors developed a retrieval plan (see Table 1). A combination of subject terms and free terms was used to search the PubMed and CNKI databases, while references were traced. The search time frame was from the establishment of the database until December 8, 2025. We utilized the database to search and analyze all literature related to keywords such as "cognitive frailty" and "elderly." Inclusion criteria included narrative reviews, systematic reviews, and clinical trials (retrospective and prospective studies). Exclusion criteria: case reports, basic research, and animal experiments. The two authors were responsible for the inclusion and exclusion of literature (see Figure 4), and in case of disagreements, the corresponding author would be consulted. A total of 75 papers were included finally.

Table 1.

Search literature summary.

Items	Details
Database	PubMed database、CNKI database
Timeline	December 08, 2025
Keywords	“older adults”,“cognitive frailty” and “influencing factors”
Inclusion criteria	Mate analysis, narrative reviews, systematic reviews, clinical trials (retrospective and prospective studies)
Exclusion criteria	Case reports, basic studies, and animal experiments
Sample size	75
Selection process	Two authors developed a search plan and performed the inclusion and exclusion of literature, consulting with a corresponding author when there was disagreement

Supplementary Table 2.

Intervention Measures	Specific Intervention Methods	Findings	Limitations
Dietary Intervention	1. Mediterranean Diet<br>2.Supplementation of enteral nutritional suspension	1. The Mediterranean diet is most beneficial for cognitive function (by increasing anti-inflammatory ω-3 polyunsaturated fatty acids)<br>2. Enteral nutritional suspension can improve the nutritional status and cognitive impairment of hospitalized patients	Purely nutritional interventions have limited effects on overall cognitive enhancement and need to be combined with other methods; additionally, the sample size of studies is small, and there are few indicators, which require further verification
Dual-task training intervention	Exercise-cognitive dual-task training	Can effectively promote cognitive function in cognitively frail elderly individuals and reduce physical frailty	Some studies did not evaluate intervention compliance; the follow-up period is insufficient ($\leq$6 months) and needs to be extended to $\geq$12 months to observe cognitive maintenance and frailty reversal rates
Hyperbaric oxygen intervention	Hyperbaric oxygen therapy (5 times a week, 110 minutes each time, for 8 weeks)	Improves MoCA score and reduces frailty degree	There are relatively few related studies; treatment duration, frequency, and regimen still require a large number of clinical trials to determine.
